# Walking, Running, Swimming: An Analysis of the Effects of Land and Water Aerobic Exercises on Cognitive Functions and Neural Substrates

**DOI:** 10.3390/ijerph192316310

**Published:** 2022-12-06

**Authors:** Laura Serra, Laura Petrosini, Laura Mandolesi, Sabrina Bonarota, Francesca Balsamo, Marco Bozzali, Carlo Caltagirone, Francesca Gelfo

**Affiliations:** 1 IRCCS Fondazione Santa Lucia, 00179 Rome, Italy; 2 Department of Humanities, Federico II University of Naples, 80138 Naples, Italy; 3 Department of Systems Medicine, Tor Vergata University of Rome, 00133 Rome, Italy; 4 Department of Human Sciences, Guglielmo Marconi University, 00193 Rome, Italy; 5 Department of Neuroscience ‘Rita Levi Montalcini’, University of Torino, 10126 Turin, Italy; 6 Department of Neuroscience, Brighton & Sussex Medical School, University of Sussex, Brighton BN1 9RY, UK

**Keywords:** physical activity, motor activity, health behaviour, brain/cognitive/neural reserve, humans, animal models, neuroplasticity

## Abstract

In the brain and cognitive reserves framework, aerobic exercise is considered as a protective lifestyle factor able to induce positive effects on both brain structure and function. However, specific aspects of such a beneficial effect still need to be completely clarified. To this aim, the present narrative review focused on the potential brain/cognitive/neural reserve–construction mechanisms triggered by different aerobic exercise types (land activities; such as walking or running; vs. water activities; such as swimming), by considering human and animal studies on healthy subjects over the entire lifespan. The literature search was conducted in PubMed database. The studies analyzed here indicated that all the considered kinds of activities exert a beneficial effect on cognitive/behavioral functions and on the underlying brain neurobiological processes. In particular, the main effects observed involve the cognitive domains of memory and executive functions. These effects appear related to structural and functional changes mainly involving the fronto-hippocampal axis. The present review supports the requirement of further studies that investigate more specifically and systematically the effects of each type of aerobic activity, as a basis to plan more effective and personalized interventions on individuals as well as prevention and healthy promotion policies for the general population.

## 1. Introduction

In the last two decades the relationship between brain damage and clinical symptoms has appeared to be more complex previously thought. Additionally, it has becoming clearer that healthy elderly individuals can recruit larger or additional brain regions than healthy young individuals to perform the same cognitive tasks [1]. Reserve mechanisms have been hypothesized as acting to make the brain more resilient to injuries by using pre-existent neural resources or by using neural and cognitive compensatory processes. In this framework, the *reserve hypothesis* has been developed, which posits that not only in aging, but also across the entire lifespan, exposure to stimulating activities is able to equip individuals with high-level resilience that can be spent in case of damage [2,3]. The *reserve* concept basically encompasses: the *brain reserve*, consisting in a very powerful cerebral structure, both at molecular and supra-molecular levels; the *cognitive reserve*, consisting in a high-level functional capacity to efficiently engage cognitive processes; the *neural reserve*, consisting in a high-level ability to recruit alternative cognitive strategies [4,5,6].

It is well known that several kinds of stimuli, including education, occupation, and leisure activities, such as cognitive, social and physical activities and exercises, contribute to the development of reserves. Namely, engagement in physical exercise has been associated with successful aging [7], and adequate motor function in the elderly has been considered as evidence of reserve occurrence [8]. Indeed, education has been found to be directly associated with walking speed and indirectly associated with white matter lesion load in the elderly [8].

In this context, the World Health Organization [9] released the global recommendation on physical activity for health, establishing a minimum level of physical activity (in terms of frequency, duration, type and total amount of exercise) during the entire lifespan. In fact, physical inactivity is the fourth risk factor for global mortality and the WHO promotes physical activity as a modifiable factor to prevent diseases and to promote general health in population. Anaerobic/aerobic exercise has been considered as an important protective factor for physical and psychological wellbeing [10]. Anaerobic activities include fast and short high-intensity exercises (such as sprinting and jumping) that do not require any oxygen consumption during their performance. Conversely, aerobic activities imply a moderate effort for a prolonged period (typical examples are walking or swimming) that allows the body to receive constant amounts of oxygen to produce energy during performance.

A large number of studies focusing on the neuroplastic effects of lifespan enriched experience have been conducted also in animal models, demonstrating that enhanced stimulations support the construction of brain/cognitive/neural reserve [11,12,13,14]. In animals, a multidimensional high-level stimulation is modeled by the experimental paradigm of environmental enrichment [15,16,17]. However, several studies have specifically focused on the analysis of the effects of physical activity in animal models, both in physiological and pathological conditions [18,19,20]. The use of rodent-based exercise models provides a number of advantages in comparison to human studies, because of their shorter gestational period and lifespan, more numerous progenies, superior genetical and physiological homogeneity, higher control in experimental procedure choices, and the larger possibility of investigation in the nervous system [21]. Moreover, rodent models provide the possibility of carrying out specific studies on the effects of anaerobic and aerobic physical activities by using specifical exercise tools and animals housed in conventional cages (without specifical exercise tools) as control subjects. Human anaerobic activities are modeled in animal resistance training designed to increase muscular strength and power and physical capacity [22]. Among the various models, an example of widely used procedures is ladder climbing, in which the animal is progressively trained to climb a ladder with a fixed load to the tail [23]. Another example is weightlifting performed by the animal standing upright with the weight typically added by means of a belt or a shoulder harness [24]. On the other hand, human aerobic activities are modeled in animals by means of voluntary or forced walking, running, and swimming (for details, see below). The effects of aerobic exercise on cognitive functions and neuroplasticity have been more widely investigated in animal models compared to studies involving anaerobic exercise [25].

On the basis of the described evidence, the aim of the present review was to offer an overview of physical activity, with particular attention to aerobic activities, as a potential builder of brain, cognitive, and neural reserves. To this aim, we considered human and animal studies on healthy subjects.

The review first presents a general section on the effects of aerobic exercise on cognitive functions and neural substrates by taking into account the evidence provided by animal and human studies. The following two sections focus on the potential brain/cognitive/neural reserve development mechanisms elicited by different aerobic exercise types: (1) land activities such as walking or running; and (2) water activities, such as swimming. Human and animal models were considered separately in these analyses.

## 2. Aerobic Exercise Effects on Cognitive Functions and Neural Substrates

### 2.1. Humans

In humans, it has been observed that aerobic exercise enhances both the mood and cognitive functioning (mainly, the executive functions) [26,27]. A recent study on young female artistic gymnasts compared with age/gender-matched sedentary children highlighted the beneficial effect of endurance and controlled aerobic training on working memory and learning abilities [28]. Studies showed that aerobic training performed for 3 days per week for at least one year limited the hippocampal decline due to normal aging processes [29,30,31,32]. Other studies showed an increase of hippocampal and entorhinal volumes after regular aerobic exercises [33,34].

Several studies have demonstrated the long-term effects of physical exercise on brain functioning, especially in the prefrontal areas and consequently on executive functions (see [35] for a review). The long-lasting beneficial effects of physical exercise on attention, working memory, cognitive flexibility, inhibition mechanisms and affective state, persisting up to 2 h after exercise cessation have also been demonstrated [35,36].

More recently, physical exercise has been found to be associated with better cognitive functioning in older adults, with increased serum neurofilament concentrations [37]. Some studies investigating the effect of different kinds of physical exercises not only on cognition [35,38] but also on the mood and emotional states, reported beneficial effects in individuals practicing sports [35,39]. Moreover, it has been shown that open skill sports (e.g., tennis) trained not only motor but also cognitive functions, in particular, inhibitory control [40,41]. Interestingly, a study investigating the effect of physical exercise on brain functional connectivity in Olympic athletes showed the strongest connections in the thalamo-sensorimotor network of the swimmers reporting the highest world rankings [42].

Recently, a study focusing on aerobic exercise associated this activity with less age-related gray and white matter loss [43]. By using functional magnetic resonance imaging (f-MRI) Vivar and colleagues [44] showed that higher-fit older adults achieved better results than low-fit adults on attentional tests. Interestingly, this finding was associated to increased BOLD signals in the prefrontal regions and by decreased signals in the anterior cingulate cortex.

Details on the original studies cited in this section are provided in Table 1.

### 2.2. Animal Models

As mentioned above, animal studies have also largely investigated the effects of physical activity on cognitive functions and neural substrates, shedding light on mechanisms able to build brain/cognitive/neural reserve [11,12,13,14]. In the bulk of studies carried out in animal models, it has been demonstrated that aerobic exercise exerts beneficial influence on cognition over the entire lifespan. Indeed, it has been demonstrated that even prenatal exposure to exercise (namely the exposure of the mothers to exercise during pregnancy) is able to improve behavioral and cognitive performance of the progeny, both in early life and in later ages [45]. Similar beneficial effects have been observed when the exposure occurred after birth, during infancy and adolescence [46], as well as during adulthood, middle age, and even aging [47,48,49,50]. Such a functional effect is accompanied by a number of neuroplastic rearrangements over the entire lifespan that involve molecular and supra-molecular processes (for a review see [51]). Many epigenetic mechanisms are believed to be involved in such structural and biological effects [52]. In particular, exposure to aerobic exercise elicits changes in the expression of neurotrophic factors and neurotransmitters [25]. Moreover, it is largely reported that a key role is played by the neurotrophin brain-derived neurotrophic factor (BDNF), which appears to regulate also the exercise-induced hippocampal neurogenesis [53].

The beneficial effects of aerobic exercise have been demonstrated in animal models with reference to several cognitive functions. To give some examples, 14 days of voluntary aerobic exercise exerted a long-lasting improvement on hippocampus-dependent memory tasks, in both female and male adult mice [54,55]. The beneficial effects of aerobic exercise beneficial effects have been shown both on short- and long-term memory performance. Alomari et al. [56] demonstrated that adult rats exposed to aerobic exercise for periods of different durations (1, 7, 14, and 28 days) showed enhanced performance both in short- and in long-term memory tasks. Such an improvement was evident after seven days of training, with no further significant enhancements after longer training periods. However, other inconsistent and not completely clear results are present in the literature. The effects of chronic voluntary aerobic exercise have been investigated at different ages in mice [57]. Animals were tested in learning and memory tasks as well as in cognitive flexibility tasks at adult age (4 months), middle age (9 months), and old age (14 months). Spatial recognition memory ameliorated in exercised middle-aged mice, but not in adult and old mice. In addition, reduced object recognition memory and cognitive flexibility were seen in exercised adult and old mice. Similarly, Segabinazi and colleagues [58] showed that adult rats previously exposed to a six-week period of aerobic training did not exhibit any improvement when evaluated for cognitive flexibility.

### 2.3. Methodology of Literature Search

Thus, although aerobic exercise has been considered as a lifestyle factor that might lead to increased physical and mental health throughout life, acting as a reserve promoter [4,59], specific aspects in such a frame need to be studied in greater depth. In particular, the present review focuses on the potential brain/cognitive/neural reserve development mechanisms elicited by different aerobic exercise types (land activities, such as walking or running, vs. water activities, such as swimming) in animal models and human beings.

For the human studies, the literature search was conducted in PubMed database, by screening the records obtained by searching for the combination of the “aerobic exercise” OR “walking” OR “running” OR “swimming” AND “cognition” OR “neural bas*” keywords. Moreover, full texts and reference lists were screened to identify further potentially relevant articles. Articles fulfilling the following criteria were included in the present overview: as population of interest, healthy human subjects of every age were included; as interventions of interest, we selected the practice of aerobic exercise, namely the practice of walking, running, or swimming; as control group of interest, we selected human subjects not practicing aerobic exercise; as outcomes of interest, we selected structural and functional effects of aerobic exercise. No language limitation was selected. No publication period limitation was selected. Records indexed up to September 2022 were screened.

For the animal studies, the literature search was conducted in PubMed database, by screening the records obtained by searching for the combination of the “aerobic exercise” OR “running wheel” OR “treadmill” OR “swimming” AND “cognition” OR “neural bas*” key-words. Moreover, full texts and reference lists were screened to identify further potentially relevant articles. Articles fulfilling the following criteria were included in the present overview: as a population of interest, healthy rodents of every age were included; as interventions of interest, we selected exposure to aerobic exercise, namely exposure to running wheels, treadmills, or swimming; as control group of interest, we selected animals not exposed to aerobic exercise; as outcomes of interest, we selected structural and functional effects of aerobic exercise. No language limitation was selected. No publication period limitation was selected. Records indexed up to September 2022 were screened.

## 3. Land Activities: Walking and Running Effects on Cognitive Functions and Neural Substrates

### 3.1. Humans

A recent study investigated brain structural and functional features in endurance runners (ER) [60]. The Authors found in the ER group compared to controls greater grey matter (GM) volumes and thicker thickness in the left precentral gyrus together with an increase of functional connectivity in the right post- and pre-central gyri [60]. Moreover, the ER group showed increased GM volume in the bilateral hippocampus and increased functional connectivity hippocampal-driven with the supplementary motor area, middle cingulate cortex, and left posterior lobe of cerebellum [60]. Finally, the ER group showed higher fractional anisotropy, an index of microstructural integrity of white matter, in the corpus callosum, left internal capsule, in the left corona radiata, and bilateral precuneus [60], suggesting thus that regular ER training impacts positively on brain structure and function.

It has also been shown that running increases neurogenesis in the dentate gyrus (DG), a part of the hippocampus that continues to proliferate during adulthood [61,62,63]. Moreover, it is well known that exercise induces high BDNF levels in serum associated with increased hippocampal volumes [30]. On the contrary, lower BDNF levels are associated with smaller hippocampal volumes as effect of aging [64]. It has been shown that physical activity impacts on grey matter volumes during aging, in particular in brain areas associated with cognition [65]. Hippocampal volumes are very sensitive to the positive effect of aerobic exercise [65]. Moreover, it was shown that the volumetric increase is lost in participants who are no longer regularly engaged in running [66]. Furthermore, after running, activation of the bilateral dorsolateral prefrontal cortex has been described in association with better executive functions [67].

From a behavioral viewpoint, the relationship between physical exercise and cognitive functioning is complicated because of several factors, such as modality, intensity and duration of the physical activity impacting on brain and cognition [68,69,70,71]. Recently, a study documented that high-intensity intermittent running enhanced executive functions in adolescents, improving their reaction times and accuracy in attentional tasks [72]. Also, long-distance marches have been reported to improve working memory performances in middle-aged athletes (age range: 40–68 years) [73].

Details on the original studies cited in this section are provided in Table 2.

### 3.2. Animal Models

Human walking and running activities may be modeled in rodents through experimental paradigms based on exposure to different kinds of voluntary and involuntary exercise, that can be scheduled to construct an acute or chronic model [74]. The exposure to *voluntary* aerobic activity is typically carried out by means of *running wheels* or an equivalent tool, such as an angled rotating running track [21]. Animal cages may be equipped with one or more wheels to provide the animals with free access to exercise; control cages are equipped with locked wheels. The minimal stress imposed to animals allows to easily realize long-term exposures. In addition, running wheels may be used as an *involuntary* exercise paradigm, by utilizing motorized wheels, which provide the researcher with the greater possibility of experimental variable controls [22]. However, the more widely used model of involuntary exercise is the *walking/running treadmill*. In this case, the researcher is able to modulate, besides the duration of the exposure, also the speed and the inclination of the apparatus in a progressive manner [75].

Animal studies focusing on *juvenile age* animals have documented the beneficial effects of the exposure to walking and running, both at functional and at structural levels, since the improvements in cognitive performance were accompanied by plastic rearrangements in the brain. A six-week exposure to free-access to a running wheel in 25-day-old rats induced the amelioration of spatial learning and memory performance, accompanied by increased hippocampal and prefrontal neuronal densities as well as vascular endothelial growth factor (VEGF) and BDNF levels. In association, anxiety levels and blood corticosterone amounts decreased [76]. A six-week exposure of 25-day-old rats to involuntary aerobic activity (treadmill; 30 min/day; 5 day/week; 8 m/min) induced similar beneficial effects, but at a lower level in comparison to voluntary activity [76]. Similarly, Merritt and Rhodes [77] documented that 30 days of voluntary wheel-running in six-week-old mice improved learning performance and increased hippocampal neurogenesis. Also, O’Leary et al. [48] reported that a seven-week period of free access to a running wheel did not change the performance of four-week-old rats in fear conditioning, but increased mRNA expression of plasticity-related hippocampal markers (including *BDNF, synaptophysin, Creb, PSD-95, Arc, TLX,* and *DCX*). As for involuntary exposure to aerobic activity, Chen et al. [78] reported that a 21-day treadmill training (60 min/day; 12 m/min) exercise regime enhanced the motor learning of four-week-old mice and increased dendritic spine formation in the motor cortex.

For *adult age* animals, substantial evidence in animal studies confirms the beneficial effects of walking and running activities in the development of reserves. Several investigations demonstrated that a long-term (one to five months) exposure to voluntary access to running wheels improved adult (two/three-month-old) rodents’ spatial learning and memory performance and increased the expression of plasticity-related hippocampal proteins, particularly BDNF [79,80,81]. In addition, Li et al. [82] reported that eight weeks of free access to wheel running ameliorated in three-month-old rats working memory performance and increased hippocampal theta activity. Motta-Teixeira et al. [83] reported that even seven days of exposure to voluntary wheel running are enough to induce hippocampal neurogenesis in two-month-old rats, though not accompanied by any improved working memory performance. As for the indication provided by the studies on forced aerobic activity with a treadmill, Li et al. [84] reported that exposure of three-month-old mice to a 28-day training (60 min/day; 10 m/min) improved learning and memory performance, and increased hippocampal neurogenesis. Wang and Wang [85] documented that eight-week-old rats exposed to different intensities (15–20–30 m/min) of treadmill training for 30 days (40 min/day) showed improved spatial memory abilities and increased neurogenesis. A caveat comes from the study by Sun and colleagues [86], who reported that seven-day high-intensity treadmill running (up to 20 m/min, imposed until the rats showed fatigue, expressed as no response to electric stimulation) may induce an impairment of spatial learning ability and hippocampal LTP, accompanied by increased hippocampal expression of hippocampal inflammatory factors in adult rats (200 g, age not specified).

Finally, evidence about reserve-building processes induced by walking and running is available also in *aging* animal models. Xu et al. [87] exposed 16-month-old mice to five months of voluntary wheel running (60 min/day; 5 days/week) and reported improved spatial learning and memory and increased density of hippocampal pyramidal cell dendritic spines. On the other hand, 30 days of involuntary treadmill running (30 min/day; 8 m/min; 5 days/week) enhanced blood volume in the motor cortex and hippocampus of 25-month-old mice [88].

Details on the original studies cited in this section are provided in Table 3.

## 4. Water Activities: Swimming Effects on Cognitive Functions and Neural Substrates

### 4.1. Humans

A recent study showed that master swimmer athletes gained successful aging in terms of better cognitive performances, better physical, psychological, and social functioning than non-athletes [89]. This research supported the association between regular aerobic exercise and reserve accumulation. Unfortunately, no brain measures were considered in this community-dwelling study [89]. Another behavioral study investigating the effect of swimming on cognition reported that the regular practice exerted a positive effect of moderation mainly on executive functioning in healthy elderly individuals [90].

Long-term swimming practice induced neural and behavioral changes in young competitive swimmers compared to novices in terms of higher motor cortical inhibition and superior sensory-motor skills when engaged in a water environment [91]. It has been shown that water immersion induces both peripheral (increased venous return and decreased antigravity muscle activity) and central responses (increased cerebral blood flow) [92,93,94,95]. Expert swimmers showed higher self-perception of passive movement sensation in a water environment than non-expert swimmers, which is likely due to higher activity in the somatosensory cortex [96]. It is well known that several brain areas, including the sensorimotor cortex, primary motor cortex, posterior parietal cortex, striatum and cerebellum are involved in sensorimotor learning. A recent study showed that sensorimotor skills of expert swimmers were superior to those observed in non-expert swimmers and that the excitability of the primary motor cortex was increased in the latter compared to the former [91].

Details on the original studies cited in this section are provided in Table 4.

### 4.2. Animal Models

The exposure of rodents to *swimming* sessions is quite easy to make. This paradigm requires a simpler tool in comparison to those needed to model walking or running. In addition, it is possible to expose a large number of animals at once to swimming sessions [75]. This experimental setting is based on the innate ability of the rodents to swim, and the control conditions can be realized by placing the animals in shallow water and maintaining the same temperature and the same duration as those used for the experimental conditions [21].

As was observed in the walking and running models, swimming animal models provided evidence that confirms neuroprotective effects over the entire lifespan, although fewer studies are available.

For *juvenile age*, four-week-old rats exposed to nine weeks of swimming regime (60–90 min/day; 5 days/week) showed improved learning abilities and attenuated accumulation of oxidatively damaged proteins [97].

Similarly, Drumond et al. [98] demonstrated that *adult* rats (150–200 g; age not specified) previously exposed to eight weeks of swimming regime (30 min/day; 5 days/week) showed improved spatial short-term memory and increased hippocampal levels of neuronal calcium sensor-1, a calcium-binding protein that regulates synaptic transmission and cortico-hippocampal plasticity, and controls learning and memory abilities and spontaneous exploration. Neuroprotective effects were found also in a six-week period of involuntary aerobic activity, obtained by exposing adult rats (180–200 g; age not specified) to alternate swimming (5 min) and resting (5 min) sessions for 60 min/day (5 days/week). By applying such a protocol, Alomari et al. [99] found improved learning and memory abilities, accompanied by increased expression of hippocampal BDNF. Moreover, by using the very same swimming protocol, Alomari et al. [56] demonstrated that even seven days of this exercise exerts enhancing effects on learning and memory abilities, with no significant further improvements following longer treatment periods (up to 28 days).

Finally, Radák et al. [97] demonstrated that swimming activity (60–90 min/day; 5 days/week; 9 weeks) significantly improved learning abilities and attenuated the accumulation of oxidatively damaged proteins even in *old* rats (14-month-old).

Details on the original studies cited in this section are provided in Table 5.

## 5. Discussion

The present review aimed to deepen the impact of aerobic exercise on cognitive functions and structural/functional brain substrates, and to demonstrate the presence of protective effects in both human and animal model studies. Such effects have been investigated with regard to the practice of both land and water activities. In particular, we considered walking, running, and swimming exercises.

On the whole, human and animal studies indicated that all these kinds of activities exert a beneficial action on cognitive/behavioral functions and on underlying neurobiological processes. In particular, the main effects observed in humans and animals involved the cognitive domain encompassing memory and executive functions, in line with previous evidence [28,35,36,54,55,56,100]. Moreover, these effects appeared to be related to structural and functional changes in several brain areas, and mainly in the fronto-hippocampal axis [60,61,62,63,79,80,81]. The reported evidence was achieved in healthy subjects and allows us to postulate that such beneficial changes constitute potential processes of brain maintaining and successful adaptation that can be spent in the case of neural damage and pathological aging. In this perspective, the evidence can be interpreted as supporting the key role of aerobic exercise in developing brain/cognitive/neural reserve [4,5]. Indeed, the beneficial effects of aerobic exercise are highly documented with regard to memory functions, which are specifically affected during aging [101]. On the whole, the human studies we considered showed the beneficial effect of aerobic exercise on memory and executive functions [10,35,101], and also on brain volume and functional connectivity involving mainly the hippocampus and prefrontal structures [60,67]. Animal studies indicated that aerobic exercise induces beneficial effects on learning and memory as well as on executive functions [56,99]. Such behavioral findings are associated with the observation of structural rearrangements, such as spinogenesis and neurogenesis, in hippocampal areas that are accompanied by changes in the expression of BDNF, which is known to be heavily involved in triggering exercise-related pathways [76,77].

A specific aim of the present review was to attempt to disentangle the peculiar effects of different kinds of aerobic exercise. Although our analysis of the literature provided a structured framework of the available evidence, the current state of knowledge does not permit us to clearly define the superiority of land or water activities in promoting healthy brain functioning. According to our literature analysis, positive effects were attested for both land and water activities, in human and animal models. These findings are inserted in a more general framework attesting the beneficial effects of aerobic activity on cognition [10,28,35,36,54,55,56]. Studies carried out on community dwelling showed a reduced risk of developing dementia in individuals practicing aerobic exercise at various levels of intensity [40]. Such evidence was provided by categorizing the different exercises on the basis of their intensity but not in terms of the specific typology. However, even by focusing the analysis on the latter criterium, we did not obtain a clear indication that supports the superiority of either typology of aerobic exercise (land or water activities). Controlled studies directly comparing the different exercises are needed to shed light on this issue. At present, some suggestions can be derived from human studies, which indicate that long-lasting aerobic training is more effective in protecting brain structure and function in comparison to intermitting or short-lasting practice [40,72]. However, animal studies suggest that even brief periods of aerobic exercise may induce some plastic rearrangement in the brain [56,83]. Additionally, indications on the beneficial effects of aerobic exercise are available at all ages of the lifespan. Specific studies differentially investigating the effects of aerobic exercise begun in childhood or later in life could provide useful indications to promote tuned and proper healthy policies for the different ages in the general population.

Despite the fact that humans practicing physical activity is a voluntary choice *per se*, animal studies provide differential indications about voluntary or forced aerobic activities. In this framework, suggestions are presently in favor of the superior beneficial effects of voluntary activity [76,86]. Such evidence suggests that a key role in the positive effects of aerobic activity is played by the motivation in engaging with it *per se*. As is well known, motivation implies reward mechanisms, which require the activation of the dopaminergic system [102,103]. It has been recently demonstrated that dopaminergic depletion plays a key role in the onset of cognitive and brain dysfunctions observed since the early stages of neurodegenerative diseases, such as Alzheimer’s Disease, both in animal [104] as well as in clinical [105,106] studies. Therefore, if aerobic exercise engages the motivational reward system and promotes an overflow of dopamine widespread in the brain, we hypothesize that it could be beneficial in both healthy subjects and those with pathological conditions for this reason alone. In particular, we argue that a dopamine overflow might mitigate the onset of cognitive symptoms in the early phases of neurodegenerative disorders. Thus, aerobic exercise may be considered as an important and accessible tool to promote healthy aging. Moreover, from this point of view it could be very interesting to investigate the effects of different aerobic activities on general psychological and emotional well-being.

However, a limitation of the present paper is that it is merely a narrative and not a systematic review based on the selection of relevant findings in relation to the topic of interest. Thus, the indications provided may be considered as suggestions and not as comprehensive summarizing findings. Future systematic studies on such an important issue appear desirable.

## 6. Conclusions

In conclusion, the human and animal studies analyzed here indicate that all the considered kinds of activities exert a beneficial effect on cognitive/behavioral functions and on the underlying brain neurobiological processes. In particular, the main effects observed involve the cognitive domains of memory and executive functions. These effects appear related to structural and functional changes mainly involving the fronto-hippocampal axis. The engagement of the motivational reward system in aerobic exercise may be involved in the beneficial effects induced by such an activity.

**Figure 1:**
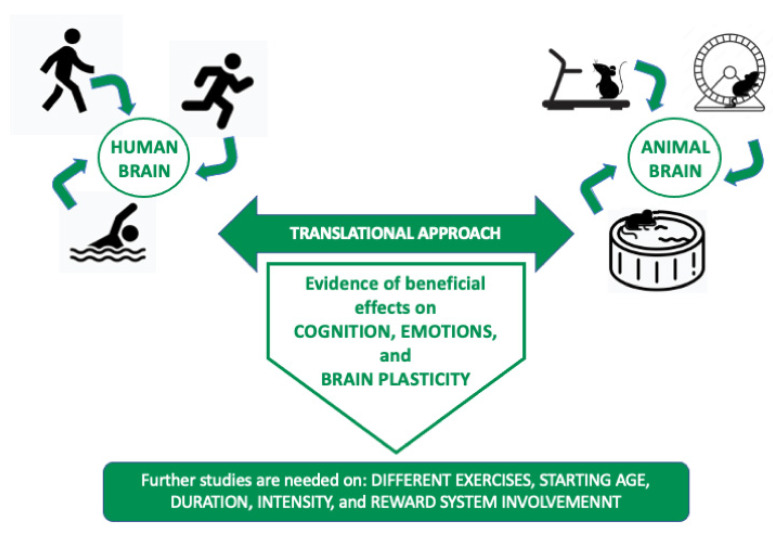
The figure illustrates the effects of the aerobic exercises investigated in the manuscript by parallelizing activities in human and animal models.

The evidence reported here and summarized in Figure 1 supports the requirement of further studies that investigate more specifically the effects of each kind of aerobic activity, as a basis in which to plan more effective and personalized interventions on individuals as well as for prevention and healthy promotion policies for the population. Such suggestions are in line with the WHO recommendations [107] affirming the importance of physical activity not only for the health of the individuals but also for the entire environment and the well-being of society. This is one of the objectives targeted in the Sustainable Development Agenda 2030 [107].

## Figures and Tables

**Table 1:** Studies on aerobic exercise effects on cognitive functions and neural substrates of healthy human subjects.

Authors	Sample Size	Design	Physical Exercises	Cognitive/Behavioural Effects	Brain Effects
**Aerobic physical activity**					
Elbaz et al., 2013 [8]	*n* = 4010; M/F% = 39%/61% Age [range] = 65–85 years	Longitudinal study over 10 years	Walking speed measured at baseline and after 4 years as 6 m by time	-	White matter lesions (deep, periventricular and total volumes assessed on structural MRI scans)
Hyodo et al., 2019 [27]	*n* = 21 M/F = 10/11 Age [range] = 65–74 years	Cross-sectional study with repeated measure	Single session: Cycling exercises (intensity = 60% of the individual ventilatory threshold; duration = 10′) vs. dance exercises (intensity = 3 different movements [twisting the upper body; swinging the arms side to side; swinging the arms back] duration = 3′.20′ for each)	Improvement in the executive functions (by Stroop task, before the training and after 5 min at the end of the training) and in the mood (vitality, stability, pleasure, arousal) (by Two dimensional mood scale before and immediately after the exercises)	-
Serra et al., 2021 [28]	*n* = 28 M/F = 0/28 Age [range] = 7–10 years	Cross-sectional study	Artistic gymnastics (Intensity = 3 sessions a week for 2 years minimum; duration = 90′ for session) vs. no gym	Improvement in the memory functions measured by the Table Radial Arm Maze task and in the schooling achievements assessed by BVN5-11	-
Erickson et al., 2009 [29]	*n* = 165 M/F = 56/109 Age [range] = 59–81 years	Correlational study	Aerobic exercises (by motor driven treadmill)	Improvement in the cardiorespiratory aerobic fitness assessed by VO_2_ peak	Increase of hippocampal volumes measured by manual segmentation on T1-weighted images obtained on 3T scan
Erickson et al., 2011 [30]	*n* = 120 Age [mean ± SD] = 66.5 ± 5.6 years M%/F% = 33.5%/66.5%	Cross-sectional study	Aerobic exercises (by motor driven treadmill; Intensity = 30–100 m/min with increments of 2% every 2 min; duration = 10′–40′ in seven weeks) vs. Stretching and toning exercise (four muscle toning exercises using dumbbells or resistance bands; two exercises for balance; one yoga sequences and one free exercise; Intensity = increasing the weight or repetitions; duration = seven weeks)	Improvement in the memory function by computerized spatial memory task preformed before, after 6 months and at the end of the trainings	Increase of hippocampal volumes measured by manual segmentation on T1-weighted images obtained on 3T scan. Scans were performed before, during and after trainings
Maass et al., 2015 [31]	*n* = 40 M%/F% = 45%/55% Age [mean ± SD] = 68.4 ± 4.3 years	Cross-sectional study	Aerobic exercises in terms of running/walking by stationary treadmills (Intensity = 30-min interval training 3 times per week; duration = 12 weeks) vs. Stretching exercise by progressive muscle relaxation (Intensity = twice a week; Duration = 45′ each session for 12 weeks)	Improvement in the recognition memory functions by the verbal learning memory test (free recall, 30-min delayed recall and recognition). Cognition was assessed before and after trainings	Increase of hippocampal volumes assessed by manual segmentation using a 7T T1-weighted images and increase in the cerebral blood flow by 3T high resolution perfusion-weighted images. Scans were performed before and after tranings
Brinke et al., 2015 [32]	*n* = 86 M/F = 0/86 Age [range] = 70–80 years	Cross-sectional study with repeated measure	Resistance Training (Keiser-based exercises; Intensity = 6–8 repetitions for two sets; Duration = 60′ daily) vs. Aerobic Training (outdoor walking program; Intensity = 40–80% of individual age-specific target heart rate; duration = 12 weeks) vs. Balance and tone Training by using stretching, motion exercises, balance exercises, functional and relaxation techniques	Improvement in the memory functions assessed by the Rey Auditory Verbal Learning test (immediate and 20-min delayed recall)	Increase of hippocampal volumes assessed by automated segmentation of 3T T1-weighted images
Whiteman et al., 2016 [34]	*n* = 33 M/F = 13/20 Age [range] = 18–30 years	Correlational study	Aerobic exercises by treadmill (Intensity = speed 0.8 m/s and incline of 10%, increasing speed of 0.35% m/sec and incline of 2% grade every 3 min; Duration = volitional exhaustion criterium	Improvement in the cardiorespiratory aerobic fitness by VO_2_ max and in memory functions by an inside/outside MRI scanner visual memory recognition test	Increase of entorhinal volumes assessed on 3T T1-weighed images by using the voxel-based morphometry approach
Chiu et al., 2017 [36]	*n* = 31 M/F = 15/16 Age [mean ± SD] = 22.2 ± 4.3 years	Cross-sectional study with repeated measure	Open skills sports (Volleyball) vs. Closed skills (Exercise) sports	Improvement in the cognitive processing speed by flanker task; improvement in aerobic physical fitness by VO_2_ max after PACER test	-
Desai et al., 2022 [37]	*n* = 1158 M/F = 430/728 Age [mean ± SD] = 77.4 ± 4.3 years	Correlational study	Physical activity assessed by US Health Interview Survey and divided in little activity (0 min of activity participation per week), medium activity (<150 min activity participation per week), high activity (>150 min activity participation per week)	Decrease of cognitive decline assessed by the East Boston Memory test; the Mini Mental state Examination and the Symbol digit modalities test. Association with neurofilament concentration was tested	-
Aly et al., 2019 [38]	*n* = 33 Age [mean ± SD] = 19.7 ± 1.5 years	Cross-sectional study	Swimming vs. Karate vs. generic physical activity assessed by the International Physical Activity Questionnaire and divided in vigorous, moderate and light intensity	Increase of attentional resources by the auditory oddball task	Changes in neurophysiological activity by EEG measurement
Zhang et al., 2022 [39]	*n* = 1117 M/F = 554/563 Age [mean ± SD] = 18.9 ± 1.2 years	Correlational study	Swimming vs. Karate vs. generic physical activity assessed by the International Physical Activity Questionnaire and divided in vigorous, moderate and light intensity	Improvement in the cardiorespiratory aerobic fitness by VO_2_ Max and decrease of negative emotional states assessed by Depression Anxiety Stress scale and by Connor-Davidson resilience scale	-
Wang et al., 2013 [40]	*n* = 60 M/F = 60/0 Age [mean ± SD] = 20.2 ± 2.9 years	Cross-sectional study	Open skills: Tennis vs. Closed skills: swimming vs. no physical activity assessed by a 7-day physical activity recall questionnaire divided in light, moderate, high, intense and sleep of PA	Improvement in the inhibitory control assessed by Stop-signal task and in the Aerobic fitness assessed by VO_2_ Max	-
Montuori et al., 2019 [41]	*n* = 27 M/F = 27/0 Age [mean ± SD] = 25.3 ± 5.2 years	Cross-sectional study	Volleyball (Intensity = 2–4 h for session 5 times a week; duration = about 13 years of experience)	Improvement in the executive functioning (Reaction time and accuracy in terms of ‘switch costs’ and errors in a switching task)	-
Huang et al., 2017 [42]	*n* = 30 M/F = 14/16 Age [range] = 20–22 years	Cross-sectional study	Swimming. Athletes were divided in high-ranked Olympic and World Championship competitors and low-ranked individuals	-	Increased thalamo-cortical functional connectivity in the sensorymotor network assessed by 3T EPI images for resting-state fMRI

Abbreviations: BVN5-11 = Batteria per la Valutazione Neuropsicologica 5-11 (Battery for neuropsychological assessment 5-11); maximal rate of oxygen consumption; EEG = electroencefalography; fMRI = functional Magnetic Resonance Imaging; MRI = Magnetic resonance imaging.

**Table 2:** Studies on land activity effects on cognitive functions and neural substrates of healthy human subjects.

Authors	Sample Size	Design	Physical Exercises	Cognitive/Behavioural Effects	Brain Effects
**Land activities**					
Cao et al., 2020 [60]	*n* = 42 M/F = 42/0 Age [mean ± SD] = 20.2 ± 2.9 years	Cross-sectional study	Endurance running (Duration = >3 years of running training) vs. no physical activity in the last 2 years	-	Increase of gray matter volume and cortical surface area in the precentral gyrus assessed by using 3T T1-weighted images; increase of functional connectivity into the same cortical regions assessed by using 3T EPI scans for resting-state fMRI; Increase of fractional anisotropy in the corpus callosum and corona radiata assessed by using 3T Diffusion weighted images
Fink et al., 2021 [66]	*n* = 48 M/F = 21/27 Age [mean ± SD] = 23.0 ± 2.2 years	Cross-sectional study with repeated measure	Running (Intensity = 5 km for 60′ session; Duration = Two-weeks running intervention time delayed: first group performed running intervention between the first T1 and the second T2 test session; the second group performed the running intervention between the T2 and the third T3 test session)	Decrease of depression levels assessed in two different tests sessions by using PANAS and CES-D	Increase of hippocampal volumes assessed by automatic segmentation on 3T T1-weighted images
Damrongthai et al., 2021 [67]	*n* = 26 M/F = 18/8 Age [mean ± SD] = 23.0 ± 2.1 years	Cross-sectional study	Running (Intensity = moderate running; Duration = single bout)	Increase in the arousal and pleasure levels by TDMS; Decrease in the interference reaction time by Stroop test. All measures assessed before and after running training	Increase activation in the prefrontal hemodynamic response by using fNIRS
Hatch et al., 2021 [72]	*n* = 38 M/F = 15/23 Age [mean ± SD] = 12.3 ± 0.4 years	Within-subject, randomized, counterbalanced, crossover design	Running trained by the LIST protocol (Intensity = 30-min high intensity intermittent running, 60-min intermittent running and rest; Duration = eight repetitions)	Increase in the executive functions (interference reaction time and attention assessed by the Stroop test, Stenberg paradigm, Flanker test)	-
Wouters et al., 2017 [73]	*n* = 521 M/F = 267/254 Age [mean ± SD] = 12.3 ± 0.4 years	Correlational study	Walking, cycling etc. assessed by SQUASH	Cognition was assessed by BAMCOG; Positive association with working memory was found	-

Abbreviations: BAMCOG = Brain Aging Monitor-Cognitive Assessment; CES-D = Center for Epidemiological Studies Depression Scale; fNIRS = functional Near-Infrared Spectroscopy; PANAS = Positive and Negative Affect Schedule; LIST = Loughborough Intermittent Shuttle Test; SQUASH = Short QUestionnaire to Assess Health-enhancing physical activity; TDMS = Two-Dimensional Mood Scale.

**Table 3:** Studies on land activity effects on cognitive functions and neural substrates of healthy animals.

Reference	Sample *(Age or Weight at the Start of Treatment)*	Kind of Exercise *(Duration)*	Effects on Cognitive Functions and Neural Substrates *(Evaluation Methodology)*
Liu et al., 2009 [79]	Male BALB/c mice (*3 months*) *n* = 10–18/group	Voluntary exercise protocol: free access to a running wheel *(4 weeks)* Involuntary exercise protocol: motorized treadmill exposure— 10 m/min^−1^; 20–60 min/day; 5 days/week *(4 weeks)*	Ameliorated spatial memory (*Morris water maze test; both protocols*); ameliorated aversive memory (*one-trial passive avoidance*; *only involuntary protocol*); increased hippocampal brain-derived neurotrophic factor (BDNF) (*ELISA*; *both protocols*), tyrosine-related kinase B (TrkB) and Syt I levels (*Western blot*; *both protocols*); increased amygdalar BDNF (*ELISA*; *only involuntary protocol*), TrkB and Syt I levels (*Western blot*; *only involuntary protocol*); transiently increased serum corticosterone levels, returned to the resting state after 24 h (*ELISA*; *only involuntary protocol*)
Lee et al., 2012 [80]	Male Wistar rats *(10 weeks)* *n* = 7–10/group	Voluntary exercise protocol: free access to a running wheel *(4 weeks)*	Ameliorated spatial learning (*Morris water maze test*); increased hippocampal BDNF protein (*Western blot*), and BDNF, TrkB, *N*-methyl-D-asparate receptor, protein kinase C mRNA expression (*quantitative RT-PCR*); unchanged plasma corticosterone levels (*radioimmunoassay*)
Li et al., 2013 [84]	Male C57BL/6 mice (*3 months*) *n* = 24/group	Involuntary exercise protocol: motorized treadmill exposure— 10 m/min; 60 min/day from 08:00 to 09:00 *(4 weeks)*	Ameliorated spatial learning and memory (*Morris water maze test*); increased hippocampal neurogenesis (*immunohistochemistry*); increased plasma corticosterone levels (*radioimmunoassay*)
Mariotti et al., 2014 [88]	Male BALB/c mice (*25 months*) *n* = 7–10/group	Involuntary exercise protocol: motorized treadmill exposure— 8 m/min; 30 min/day; 5 days/week *(4 weeks)*	Increased cerebral blood volume in hippocampus and motor cortex; unchanged cortical thickness (*magnetic resonance imaging—by using a Biospec Tomograph equipped with a 4.7 T 33 cm bore horizontal magnet*)
Merrit and Rhodes, 2015 [77]	Male mice of different strains-B6, 129S1, B6D2F1, D2, and B6129F1 *(6 weeks)* *n* = 10–11/group	Voluntary exercise protocol: free access to a running wheel *(4 weeks)*	Ameliorated spatial learning (*4-arm plus water maze test*); increased hippocampal neurogenesis (*immunohistochemistry*)
Uysal et al., 2015 [76]	Male and female Wistar rats *(28 days)* *n* = 7/group; M/F 1:1	Voluntary exercise protocol: free access to a running wheel *(6 weeks)*	Ameliorated spatial learning and memory (*Morris water maze test; both protocols*); decreased anxiety levels (*open field test*; *Morris water maze test; both protocols for males, but only voluntary protocol for females*); increased hippocampal and prefrontal neuronal densities (*cresyl violet staining; both protocols*) increased vascular endothelial growth factor (VEGF) and BDNF levels (*immunohistochemistry; ELISA*; *both protocols*); decreased serum corticosterone levels (*not specified method*; *only voluntary protocol*)
		Involuntary exercise protocol: motorized treadmill exposure— 8 m/min; 30 min/day; 5 days/week *(6 weeks)*	
Motta-Teixeira et al., 2016 [83]	Male Wistar rats *(2 months)* *n* = 27–28/group	Voluntary exercise protocol: free access to a running wheel *(7 days)*	Unchanged working memory performance (*Morris water maze test*); increased hippocampal neurogenesis (*immunohistochemistry*)
Venezia et al., 2016 [81]	Male and female C57Bl/6J mice *(8 weeks)* *n* = 10/group; M/F 1:1	Voluntary exercise protocol: free access to a running wheel *(20 weeks)*	Increased hippocampal *Bdnf IV* (*quantitative RT-PCR*) and total *Bdnf* mRNA (*quantitative RT-PCR*; *only in males*) expression; increased hippocampal mature BDNF protein expression (*ELISA; only in males*)
Wang and Wang, 2016 [85]	Female Wistar rats *(8 weeks)* *n* = 10/group	Involuntary exercise protocols: motorized treadmill exposure— 15–20–30 m/min; 40 min/day *(30 days)*	Ameliorated spatial memory performance (*Morris water maze test*); increased hippocampal neurogenesis (*immunohistochemistry*)
Sun et al., 2017 [86]	Male Sprague Dawley rats (*200 ± 20 g*) *n* = 8–16/group	Involuntary exercise protocols: motorized treadmill exposure— 20 m/min; imposed until the rats showed fatigue, expressed as no response to electric stimulation *(7 days)*	Ameliorated spatial learning performance (*Y-maze active avoidance test*); suppressed induction and maintenance of hippocampal LTP (*evocation and recording of the field excitatory postsynaptic potentials*); increased hippocampal IL-1β, TNF-α, and iNOS expression (*semiquantitative PCR*); upregulated hippocampal levels of phosphorylated JNK (p-Jnk), phosphorylated p38 (p-p38), and phosphorylated ERK (p-Erk) (*Western blot*)
Xu et al., 2017 [87]	Male C57BL/6J mice *(16 months)* *n* = 12–20/group	Voluntary exercise protocol: free access to a running wheel *(60 min/day; 5 days/weeks; 5 months)*	Ameliorated spatial learning and memory performance (*Morris water maze test*); increased density of hippocampal pyramidal cell dendritic spines (*Golgi staining*); prevented age-related loss of hippocampal postsynaptic density protein-95 (*immunohistochemistry*)
Chen et al., 2019 [78]	Male C57BL/6J and Thy1-YFP transgenic mice *(4 weeks)* *n* = 4–8/group	Involuntary exercise protocols: motorized treadmill exposure— 12 m/min; 60 min/day *(3 weeks)*	Ameliorated motor learning performance (*rotarod test*);in motor cortex: increased mature BDNF protein, TrkB, phosphorylated mTor, phosphorylated ribosomal S6 protein, postsynaptic protein PSD95, vesicular protein SNAP25 expression (*Western blot*); inhibited elongation factor 4E-BP2 (*Western blot*); increased postsynaptic density lengths and thicknesses (*electron microscopy*); elevated the amplitude of miniature excitatory postsynaptic current in layer 5 pyramidal neurons (*whole-cell patch-clamp recording*); increased dendritic spine formation in layer 3 and 5 pyramidal neurons (*in vivo imaging*); increased axonal myelination (immunohistochemistry; *electron microscopy*)
O’Leary et al., 2019 [48]	Male Sprague Dawley rats *(4/8 weeks)* *n* = 7–11/group	Voluntary exercise protocol: free access to a running wheel *(7 weeks)*	Increased hippocampal-dependent learning (*fear and cued fear conditioning test*; *only after training initiated at 8 weeks of age*); increased hippocampal *BDNF, synaptophysin, Creb, PSD-95, Arc, TLX,* and *DCX* mRNA expression (*quantitative RT-PCR*; *only after training initiated at 4 weeks of age*); increased amygdalar synaptophysin mRNA expression (*quantitative RT-PCR*)
Li et al., 2021 [82]	Male Wistar–Kyoto rats *(12 weeks)* *n* = 6–12/group	Voluntary exercise protocol: free access to a running wheel *(8 weeks)*	Ameliorated working memory performance (*8-arm radial maze test*); increased middle to high range frequency (6.5–12 Hz) of hippocampal theta power (*24-h electrophysiological recording*)

Note: unless otherwise specified, the described effects regard all the exercised groups.

**Table 4:** Studies on water activity effects on cognitive functions and neural substrates of healthy human subjects.

Authors	Sample Size	Design	Physical Exercises	Cognitive/Behavioural Effects	Brain Effects
**Water activity**					
Abou-Dest et al., 2012 [90]	*n* = 48 M/F = 27/21 Age [mean ± SD] = 23.5 ± 2.5 years; 69.1 ± 3.8; 69.2 ± 3.2	Cross-sectional study	Swimming (Duration = at least twice a week for at least 2 years)	Increase in the executive functions assessed by SRT, CRT, Stop-signal task and Stroop test	-
Sato et al., 2020 [91]	*n* = 28 M/F = 18/10	Cross-sectional study	Swimming	-	Positive association with intracortical inhibition in motor cortex assessed by TMS before and after water immersion
Carter et al., 2014 [94]	*n* = 9 M/F = 9/0 Age [mean ± SD] = 24.6 ± 2.0 years;	Cross-sectional study with repeated measures	Water immersion	-	Increase in cerebral blood flow on middle and posterior cerebral artery by using a 2-MHz pulsed ST3 transcranial ultrasound system
Sato et al., 2013 [95]	*n* = 15 M/F = 15/0 Age [mean ± SD] = 21.7 ± 0.4 years;	Cross-sectional study with repeated measures	Water immersion	-	Changes in sensorimotor integration assessed by TMS before during and after 15 min water immersion

Abbreviations: CRT = visual 2-choice Reaction Time test; SRT = auditory Simple Reaction Time; TMS = Transcranial Magnetic Stimulation.

**Table 5:** Studies on water activity effects on cognitive functions and neural substrates of healthy animals.

Reference	Sample *(Age or Weight at the Start of Treatment)*	Kind of Exercise *(Duration)*	Effects on Cognitive Functions and Neural Substrates *(Evaluation Methodology)*
Radák et al., 2001 [97]	Male Wistar rats *(4 weeks/14 months)* *n* = 12–20/group	Swimming–60 min/day; 5 days/week *(6 weeks);* then, 90 min/day; 5 days/week *(3 weeks)*	Ameliorated active avoidance conditioning (*pole-jumping conditioned avoidance behavior test*); ameliorated short- and long-term memory (*passive avoidance behavior test; only in 14-month-old rats*); decreased brain protein carbonyl levels (*spectrophotometric measurement and Western blot*); increased brain chymotrypsin-like activity of proteasome complex (*fluorometric measurement*); increased brain DT-diaphorase activity (*spectrophotometric measurement; only in 4-week-old rats*)
Drumond et al., 2012 [98]	Male Wistar rats *(150–200 g)* *n* = 5–15/group	Swimming–30 min/day; 5 days/week– with 60% of maximal supported load, previously determined *(8 weeks)*	Ameliorated spatial short-term memory (*object location test*); increased hippocampal levels of Neuronal Calcium Sensor-1 (*Western blot*)
Alomari et al., 2013 [99]	Male Wistar rats *(180–220 g)* *n* = 14–15/group	Swimming/rest protocol 5 min alternatively for 60 min/day; 5 days/week *(6 weeks)*	Ameliorated spatial learning and short- and long-term memory (*radial-arm water maze test*); increased hippocampal brain-derived neurotrophic factor (BDNF) levels (*ELISA*)
Alomari et al., 2021 [56]	Male Wistar rats *(180–220 g)* *n* = 12/group	Swimming/rest protocol 5 min alternatively for 60 min/day; 5 days/week *(0, 1, 7, 14, or 28 days)*	Ameliorated spatial learning and short- and long-term memory (*radial-arm water maze test*; *only after 7, 14, and 28 days of exercise*)

Note: unless otherwise specified, the described effects regard all the exercised groups.

## Data Availability

The concept reported in this manuscript is not associated with raw data.
